# Can’t bibliometric analysts do better? How quality assessment without field expertise does not work: A comment on G. Madison and T. Söderlund: Comparisons of scientific quality indicators across peer-reviewed journal articles with more or less gender perspective: Gender studies can do better

**DOI:** 10.1007/s11192-018-2872-x

**Published:** 2018-08-03

**Authors:** Nina Lykke

**Affiliations:** Department of Thematic Studies: Gender Studies, Linköping University, Linköping, Sweden

**Keywords:** Gender studies, Quantitative quality assessment, Differences between qualitative and quantitative research, The importance of field specific knowledge

## Abstract

The article is an invited comment on Guy Madison and Therese Söderlund (M&S): Comparisons of content and scientific quality indicators across peer-reviewed journal articles with more or less gender perspective: Gender studies can do better. *Scientometrics* 115(3):1161–1183. The article pinpoints a series of serious problems in M&S’s quantitative quality assessment and analysis of the field of gender studies, pertaining to their overall conceptual framework, their general approach and their specific analysis. It is argued that the over-arching problem in M&S’s study is their lack of expert knowledge of the field of gender studies, their lack of respect for differences between qualitative and quantitative research, and their research design, which is biased towards quantitative social and natural science research. Firstly, it is demonstrated that a key concept, ‘gender perspective’, is used in an incoherent and confusing way in M&S’s analysis. Secondly, it is argued that the confusion is not an isolated definitional problem, but related to a series of slippages between M&S’s source of inspiration (Ganetz in Genusvetenskapliga projektansökningar inom humaniora-samhällsvetenskap – en uppföljning av Vetenskapsrådets beredning och utfall år 2004. Vetenskapsrådets rapportserie, Stockholm 15/2005, 2005) and their own adoption of the category. Thirdly, differences between qualitative and quantitative research, and between hermeneutic and explanatory knowledge production, are discussed more broadly to sustain the argument that the mentioned slippages occur, because M&S transfer analytical tools from Ganetz’ qualitative study, based on a peer review methodology, to a quantitative quality assessment, carried out without field specific expert knowledge. It is argued that, to be adequate and relevant, a quality assessment would need to respect these differences, and develop tools and research designs accordingly. Fourthly, the validity of M&S’s content analysis—the core of their study—is questioned in detail because of its use of inadequate analytical categories, and because of its exclusion of central elements from the analysis. Finally, it is argued that the bias in M&S’s research design is reproduced in their results.

I have been invited by the editors of *Scientometrics* to comment on the above mentioned article of Madison and Söderlund (2018), and, due to my expertise in the area, which Madison and Söderlund (2018) has submitted to bibliometric analysis, I accepted the invitation. I welcome that the editors of *Scientometrics* by this invitation demonstrate that they find it important to consult a scholar with expert knowledge in the area of scholarly knowledge production under scrutiny—confirming it as a principle for bibliometrics that ‘precise knowledge about the examined scientific domain [is] required to carry out and interpret bibliometric investigations correctly’ (Wallin 2005: 261). I am happy to learn that the editors endorse this principle, in particular because one of the problems of Madison and Söderlund (2018) is that its authors allegedly disregard it.

The analysis, carried out by Madison and Söderlund (in the following referred to as M&S), is based on the assumptions: (1) that Gender Studies can be defined as a bounded object to be quality assessed through a quantitative analysis; (2) that a quality assessment of an academic field of research can be carried out without involving expertise from the field in question. In my comments, I shall discuss the consequences of M&S’s rejection of the need to reflect on these two assumptions, and, against this background, demonstrate how a series of conceptual and methodological problems undermine the validity of their analysis.

## M&S’s aim and assumptions

Let me start noting that the diversity of the field of gender studies is acknowledged in M&S’s article introduction. But criticisms vis-à-vis gender studies are nonetheless reported by M&S, as if they addressed a bounded, consensual object, and represented a consensual line of critique. Criticisms by scholars from outside the field (e.g. Rothstein 1999; Popova 2005; Söderlund and Madison 2015, 2017) are confounded with critical analyses by academic feminists who identify with the field, and who, through their criticism, demonstrate the field’s non-consensual character, its diversity and ability to develop through controversies (e.g. Friedman 1997; Pereira 2012). However, against the background of the assumption that the area is so well bounded that it can be evaluated en bloc through a quantitative analysis, and that the criticisms are so much in resonance with each other that their relevance (or lack thereof) can be assessed jointly, M&S state it as their article’s aim to empirically investigate and assess, whether or not there is a well founded basis for the critique. The authors state it as their aim to carry out such an assessment through a quantitative analysis, specifically based on a meta-analysis of a sample of 36 articles, [dealing with questions ‘related to gender and sex’ (Madison and Söderlund 2018: 1164)]. The articles are randomly selected from the authors’ earlier compiled database (Swedish Gender Studies List, SGSL, covering the period 2000–2011) (Söderlund and Madison 2015), so that the sample includes three types of articles (12 of each type), taxonomized by M&S in terms of their manifestation of ‘more, less and no gender perspective’ (2018: 1161).

Using SGLS as foundation for the selection of the sample of 36 articles, M&S implicitly demonstrate that they do not take into account earlier critiques of their methodology, used in the construction of SGSL (Lundgren et al. 2015). They implicitly confirm what they, in response to this criticism, have stated earlier (Madison and Söderlund 2016: 331): that they ‘do not accept’ that the complexity of the transdisciplinary field of gender studies should make it impossible to study ‘systematically and quantitatively’:

Gender Studies is indeed more difficult to define and work with than any other body of research that we have previously addressed in meta-analyses or systematic reviews, and the elaborate search criteria and multiple sources employed attest to our ambition to maximize validity. LSL’s [reference to Lundgren, Shildrick and Lawrence 2015, NL] despondent statements seem to suggest that it cannot therefore be studied systematically and quantitatively, an implication we do not accept. (Madison and Söderlund 2016: 331).

So M&S’s point of departure is a firmly affirmative answer to the question ‘Can gender studies be studied?’ [the title of M&S’s response to the critique of the conceptual foundations for SGSL (Madison and Söderlund 2016)].

Through the approach used in the present article, M&S also implicitly confirm another statement, which they made explicit earlier (Madison and Söderlund 2016), namely that expertise in the field under scrutiny is not necessary for carrying out a quantitative meta-analysis; in their response from 2016, M&S even state that it potentially would be harmful to scientific ideals to argue otherwise:

Their [LSL, reference to Lundgren et al. 2015, NL] claim that “a research team without any expertise within the field” cannot “properly conduct a bibliometric study” seems quite dubious: LSL [reference to Lundgren et al. 2015, NL] seem in effect to be saying that the dissemination and impact of this “way of thinking” [Gender Studies, NL] can only be studied by those who think this way. That would challenge central scientific ideals, such as replicability and communalism (Merton 1973; Ziman 2000). (Madison and Söderlund 2016: 331)

I shall not go further into the debate around Söderlund and Madison’s 2015-article. The critique of it was already elaborately spelled out by Lundgren et al. (2015). I insert the above quotes from M&S’s earlier work (2016) to underline that the problematic assumptions, which I, as mentioned above, shall discuss in relation to the present article, and which are more implicitly stated here, have earlier been explicitly and rather emphatically articulated by M&S.

## Incoherent framing of results

I shall first comment on M&S’s framing of their main results in order to document the conceptual incoherence which their insufficient knowledge and understanding of the field of gender studies generate. As summarized in M&S’s abstract, a main conclusion of their study of the 36 articles on gender and sex is that ‘the higher level of gender perspective, the lower was the scientific quality for seven out of nine indicators.’ (Madison and Söderlund 2018: 1161). Given that M&S claim that their approach and definition of ‘gender perspective’ is ‘inspired by Ganetz (2005)’ (Madison and Söderlund 2018: 1165), this framing of the main conclusion appears as logically incoherent and contradictory. To make this incoherence visible to readers who are not familiar with Ganetz’ analysis, I shall provide a bit more detail (as it was done also in the comment by Lundgren, Shildrick and Lawrence who criticized M&S for a ‘distortion’ of Ganetz’ taxonomy (Lundgren et al. 2015: 1391)—a critique which M&S apparently have chosen to ignore, in so far as they continue to use Ganetz’ work inappropriately in the present article).

In a meticulous investigation of the degree of integration of gender perspectives in research applications to the Swedish Research Council, commissioned and published by the Gender Studies Committee of this Research Council, Ganetz (now professor of Gender Studies at Stockholm University) defines three levels of integration: ‘gender studies’, ‘gender perspective’ and ‘gender aspects’ (Ganetz 2005: 12–14).^1^ The definition is described as ‘operative’ (Ganetz 2005: 12), i.e. not intended as a definition to be applied generally (as M&S seem to imply, 2016: 332), but specifically related to an empirical study of the ways in which applicants to the Swedish Research Council in 2004 by ticking (or not ticking) a ‘gender box’ indicated whether or not their project applications, in their own opinion, addressed ‘questions concerning gender/gender perspective’ (Ganetz 2005: 6). Ganetz’ first two taxonomical categories, ‘gender studies’ and ‘gender perspective’, are, in her definition, characterized by knowledge and understanding of the field, i.e. ‘shared frames of reference’ in terms of knowledge and understanding of ‘theories and methodologies’ as well as ‘empirical approaches’ and ‘research traditions’, developed within ‘the big field of knowledge production which problematizes how gender, i.e. cultural and social gender, is “done”, constructed, materialized, formed’ (Ganetz 2005: 12–13). Ganetz’ third category, ‘gender aspects’, is characterized by a more superficial and weak knowledge and understanding of the field (Ganetz 2005: 13), and is in practice sometimes confounded with the simple use of the categories women and men as ‘variables’: according to Ganetz’ definition, the latter does not in itself qualify a research project to be counted under the tickbox umbrella ‘questions concerning gender/gender perspective’. (Ganetz 2005: 13–14).

As mentioned, M&S claim that the taxonomy, on the basis of which they constructed SGSL as well as the sample of 36 articles (selected from SGSL) to be submitted to quality assessment—namely ‘self-identified’, ‘inferred’ and ‘neutral’—is ‘inspired by Ganetz (2005)’ (2018: 1165–1166). As already emphasized by Lundgren et al. (2015: 1391), what happened in M&S’s earlier work was a ‘distortion’ of Ganetz’ definitions and approach. In the new study, this distortion leads to an incoherent framing of results, and, as I will elaborately discuss in the next section, unacknowledged slippages which seriously problematize the validity of the study.

However, let me first demonstrate the problems by asking what it does to the logic of M&S’s argument, if we take their interpellation of Ganetz’ taxonomy at face value—which their use of the term ‘gender perspective’, combined with the centrally located reference to Ganetz’ work, indeed, invites their readers to do.

Seen against the background of Ganetz’ definitions, M&S’s framing of their main conclusion as ‘the higher the level of gender perspective, the lower was the scientific quality’ (2018: 1161) amounts to stating that when studying gender and sex, the higher a level of scholarly quality can be reached, the less knowledge, understanding and overview the researcher possesses regarding research traditions, theories and methodologies, used in the field. This is, indeed, a rather absurd statement, challenging basic scientific ideals about knowledge, understanding and overview of previous research and research traditions as an important requirement in all research!

## Inappropriate use of theoretical sources

The unacknowledged incoherence, pointed out above, indicates that M&S either misunderstand Ganetz’ work, or that they, without making it clear to their readers, twist it for their own purposes. Whatever is the case, M&S replace Ganetz’ definition and taxonomy with their own, which decisively differs from Ganetz. It is, therefore, not only inconsequent, when they use Ganetz’ terminology, when framing their main conclusion; it is also scholarly inappropriate to do so without indicating the decisive differences. M&S do, indeed, postulate that the methodology, which they developed in this article in order to carry out their quality assessment, takes into account the critique raised by Lundgren et al. (2015), which among others addressed their use of Ganetz’ terminology and approach (Madison and Söderlund 2018: 1164). However, M&S continue to ignore the decisive differences between their alleged inspiration, Ganetz (2005), and their own work, and this results a series of crucial slippages, which I shall spell out in terms of the way they operate in the present article.

But what are the differences between M&S’s and Ganetz’ taxonomies, approach and definitions?

M&S’s approach and use of Ganetz’ work can be summarized as follows: When they, as mentioned, claim that the taxonomy (’self-identified’, ‘inferred’ and ‘neutral’), on the basis of which they construct their sample of 36 articles to be analysed, is ‘inspired by Ganetz (2005)’, they sustain this claim by referring to their taking into account ‘both self-identification and subject matter’ of the selected articles (2018: 1165). Moreover, M&S redefine Ganetz’ term ‘gender perspective’, the entity, which they ‘postulate’ as ‘characteristic of gender studies publications’ and possible to ‘quantify’, as ‘a relatively coherent set of values and beliefs, epistemological and other’ (2018: 1164). Moreover M&S claim to be able to carry out a quality assessment through a mechanic quantification of the degree to which the ‘gender perspective’, defined as these values, are present (or absent) in a given article, and to do so without involvement of field-specific expert knowledge.

In relation to Ganetz’ definitions and methodology, a series of crucial, but unacknowledged slippages can be noted.

Firstly, Ganetz’ main criteria, when evaluating the level of integration of gender studies/gender perspective/gender aspects in applications to the Swedish Research Council, is, as mentioned, whether or not the applicants demonstrate knowledge, understanding and overview of the field in terms of theories, methodologies, empirical approaches, and research traditions. In other words, Ganetz’ focus is neither a fixed set of values, nor a bounded list of subject matters. One key slippage here relates to the ways in which M&S shift Ganetz’ definition of ‘gender perspective’ from a register concerned with knowledge and understanding of the field to one concerned with values. Through an unacknowledged change of discoursive registers (from knowledge to values), it seems as if M&S, through a biased framing, prepare for the conclusion that gender studies is based on ideological values and not on scholarly knowledge, and in this sense not living up to normal scientific standards.

A second slippage, closely related to the first one, concerns the ways in which M&S—by contrast to Ganetz—impose a specific definition of subject matter on the material to be analysed. The question of subject matter is included in M&S’s analytical framework in order to enable them to assign the articles in their material to one of three article categories, ‘self-identified’, ‘inferred’, and ‘neutral’, and subject matter is specified as (1) acknowledgement of ‘an uneven power relation between men and women’, (2) considering ‘sex as socially and culturally constructed’ and/or (3) focus on ‘injustices and discrimination based on gender, race, ethnicity, sexuality, age, religion and disability’ (Madison and Söderlund 2018: 1166). M&S take these definitions of subject matter from the *Swedish National Encyclopedia*. Though, by contrast to M&S, the Encyclopedia aligns itself with terminology commonly used in the field of gender studies, speaking, for example, of social and cultural construction of ‘gender’ instead of M&S’s subject matter category ‘sex as socially and culturally constructed’ (Madison and Söderlund 2018: 1166). However, the main problem to be noted here is neither issues of terminology nor the definitions per se, but the very act of operating with fixed subject matter categories, which makes M&S’s methodology for assigning articles to one or the other category unflexible, and not well suited to address a diverse field. Following a strong research tradition for inclusiveness and open-endedness in the field of gender studies, Ganetz does not refer to bounded subject matters, when distinguishing between the level of gender perspective in the applications, she analysed. The open-endedness as regards subject matter, which Ganetz’ focus on knowledge and understanding allows, enables her to take into account that gender studies is a field, which does not seek consensus, and where researchers working within the framework of the umbrella ‘Gender Studies’, for example, as employees of university centres or departments of Gender Studies (which seem to make up the authors of a major part of the articles in M&S’s category ‘self-identified’), have very diverging approaches to ‘gender’ (Lundgren et al. 2015; for an elaborate discussion, see Lykke 2010, 2011, 2016). When sliding away from Ganetz’ open-ended and flexible framework into using fixed subject matter categories, M&S construct an analytical framework, which is not adequate in relation to the field they want to study.

I shall note here, that M&S, in the reply to Lundgren et al. (2015), M&S argue that this kind of problematization of the foundation of the overall self-identified article group in SGSL is not relevant, in so far as their use of Ganetz’ classification primarily intended to ‘separate non-self-identified publications that contained gender perspective from those that did not’ and that ‘this did not at all affect the self-identified publications, but did only divide the Neutral category’ (Madison and Söderlund 2016: 331). However, as M&S explain their research design in the new article (2018), the subject matter definition has, as described above, serious implications for the assignment of articles to the ‘self-identified’ and ‘inferred’ article groups.

A third slippage concerns the category ‘self-identification’. In M&S’s analysis, this category is imposed on the article-material by the researchers; by contrast to Ganetz they put a procedure to work, which literally cannot be described as ‘self-identification’. As mentioned, Ganetz is studying an empirical material of applications, where applicants are asked to perform or abstain from performing an act of ticking a box, regarding ‘gender/gender perspective’ (Ganetz 2005: 12). In Ganetz’ case ‘self-identification’, thus, relates to an active act, the ticking or not ticking of a box as part of an application procedure, related to a specific research project. In the study, carried out by M&S (2018), there is not such an active act of self-identification involved specifically in relation to the articles in the sample, which evidently opens for more arbitrariness than in Ganetz’ study. When, in addition, the assignment of articles into the ‘self-identified’, ‘inferred’ and ‘neutral’ article-groups in M&S’s study is undertaken by scholars without expert knowledge in the domain of gender studies, the space for arbitrary assignments is left wide open.

Finally, it is a notable slippage in relation to Ganetz’ approach that she, by contrast to M&S, did not develop her analytics with the purpose of carrying out a mechanical quantitative analysis. Her categories and approach were instead developed to be made operative in a qualitative assessment of field-specific knowledge and understanding, demonstrated in project proposals. In other words, Ganetz’ analytics, and her way of working, was in line with the principles and methodologies of peer reviewing. It was a quality assessment, based on a qualitative analysis of specific kinds of knowledge and understanding demonstrated in scholarly texts (in casu: submitted research applications). This was an assessment which Ganetz, in line with the principles and methodologies of peer reviewing was qualified to undertake qua her expert knowledge in the field, which also was the reason why she was commissioned by the Gender Studies Committee of the Swedish Research Council to carry out the assessment.

In their response to Lundgren et al. (2015), M&S claim that ‘Ganetz’ criteria can be applied with reasonable accuracy by non-experts, which is indeed what they were developed for.’ (Madison and Söderlund 2016: 331–332). The series of slippages, documented above, demonstrates the inadequacy of this claim.

## Lack of acknowledgement of difference between research fields

So far I put focus on problems regarding M&S’s use of conceptual frameworks, analytic categories and approaches. I shall proceed to questions concerning the overall methodology for quality assessment, chosen by M&S. M&S make it clear from the start that their purpose is to assess the criticisms, raised in relation to gender studies, against the background of ‘commonly accepted quantitative indices’ (Madison and Söderlund 2018: 1162). Moreover, they state that the ‘fundamental aspects of scientific quality’ which they aim to analyse, are ‘the possibility to draw inferences’, ‘the generalisability and validity of results’ and ‘accounting for limitations with the authors’ own work’ (Madison and Söderlund 2018: 1163). What M&S state that they want to carry out is, in other words, a quantitative analysis, based on quality criteria with a certain bias towards quantitative social and natural sciences [cf. ‘generalisability’ and to some extent ‘validity’ (Olesen 2000: 230)]. In the discussion section at the end of M&S’s article, it is also clearly stressed that the criteria, against the background of which the quality of the 36 articles in the sample are measured, are biased towards quantitative natural and social sciences. M&S conclude, for example, that their analysis indicates that ‘gender studies has positioned itself more distantly from *the* scientific method’ (2018: 1178) (my italics, NL)—a method, which, as stressed in the next sentence, is quantitative, putting ‘generalisability’ central (ibid), and which it is claimed to be problematic to distance oneself from: ‘It may therefore be argued that gender studies could benefit from implementing established research methods employed in other disciplines’ (ibid.).

The approach to scholarly quality assessment, which M&S articulate here, ignores the longstanding debates on differences within the field of what in German and Scandinavian languages with an overall term is called ‘Wissenschaft’/’vetenskap’, while in English dichotomized as ‘arts’ and ‘sciences’, hermeneutic and explanatory fields of scholarly knowledge production [dating back to classic contributions of German philosopher Wilhelm Dilthey 1833–1911 (Dilthey 1991)]. Without even mentioning these debates and differences, M&S measure gender studies on criteria which do not fit the kind of research and knowledge production, which has been most widespread within the field, namely qualitative and interpretative research. Perhaps in an attempt to counter-act an argument like the one I am unfolding here, regarding methodological differences, M&S argue that ‘the question of fairness misses the point’ (2018: 1177). However, what I am bringing to the fore here is certainly not a discussion of ‘fairness’, but a question of choosing methodologies and assessment criteria which are appropriate for the material to be studied. Moreover, this is a not a question concerning only gender studies, but one that relates to another mainstream in knowledge production than quantitative social and natural science research, namely the kinds of scholarly knowledge production, characterizing the humanities and those parts of the social sciences, which have a preference for qualitative, interpretative research. Let me illustrate the problem of evaluating and measuring research quality in these areas through quality criteria, relevant for quantitative natural and social science research, with a thought experiment: What sense would it make to evaluate research within the field of philosophy for its lack of empirical testing? Or to evaluate studies of literature and visual arts for their lack of representativity and generalisability? What M&S (2018) apparently aim to do in terms of evaluating an assumed majority of articles on qualitative social science and humanities research with the tools and criteria of quantitative social and natural science research amounts to the same kind of absurdity.

But not only do M&S gloss over these historical debates and qualitative differences between areas of scholarly knowledge production, it also seems as if they more specifically lack in-depth knowledge of qualitative research; at least they claim that ‘there can be no opposition between analyzing qualitative data and considering their reliability, validity, and representativity, as well as causal relations amongst constructs’ (Madison and Söderlund 2018: 1178). This claim indicates that the authors must be unfamiliar with the specificities of qualitative research, and its marked differences from quantitative research regarding among others representativity, which is not sought for in qualitative research (Denzin and Lincoln 2000: 8).

I agree in principle with M&S, when they claim that qualitative and quantitative research may be combined, and that the two approaches can mutually enrich each other, when they address a joint topic, and jointly create a more complex analysis than each of them can generate independently. However, to create a mutual enriching relationship between quantitative and qualitative research will require that the qualitative differences between the approaches are taken carefully and systematically into account—as appropriately illustrated by the pluralistic and non-hierarchical use of both quantitative and qualitative research methods, suggested by Guest Professor of Gender and Neuroscience, Gillian Einstein, Linköping University and University of Toronto (Einstein 2012), in a study of female genital cutting, informed by feminist approaches to neuroscience. This is an approach where neither the quantitative nor the qualitative approach is setting the overall agenda, but where continuous transversal dialogues regarding research questions to pursue and methods to be used are taking place. This is an approach, which is decisively different from the one defended by M&S, where the quantitative approach appears as ‘the scientific method’ par excellence (Madison and Söderlund 2018: 1178).

To avoid the bias towards quantitative social and natural sciences, and adequately address the above differences between knowledge production, based on qualitative and quantitative approaches (differences, which I assume are reflected in the sample of 36 articles, analysed by M&S, even though I, of course, cannot know for sure, since I do not have access to M&S’s list of articles), it would have been more appropriate to carry out a quality assessment against the background of a full peer review of all 36 articles. Given the relatively small sample of articles, the choice of quantitative analysis, cannot be defended by numbers. To qualitatively peer review 36 articles would not have been an impossible task!

## Content analysis without content

Having highlighted problems regarding conceptual framework, use of theoretical sources, and general approach to methodologies and differences between quantitative and qualitative research, I shall, as my final comment, zoom in on the specifics of M&S’s analysis, and focus on some of its key problems.

Firstly, along the lines of the problematic use of assessment methods, discussed above, it should, more specifically, be noted that the content analysis, on which the analysis is based, is limited to statements in sections of the 36 articles under scrutiny, ‘labelled as background, discussion, introduction, and overview’, while ‘sections describing the aim, material, method, or results’ are left out (Madison and Söderlund 2018: 1165). The argument for this choice is that it is the group level, and not the specifics of the individual articles which is the focus of the investigation. But this way of delimiting the material for the content analysis adds to the bias of M&S’s article. Precisely the sections, which are left out, would be crucial for an evaluation of qualitative social science and humanities research, often working with emerging methods, closely related to research design and materials. It is bound to seriously undermine the validity of the results of the quality assessment, if these sections are not taken into account vis-à-vis an article sample which presumably includes qualitative social science and humanities research. For example, given the ways in which qualitative social science and humanities gender studies articles are often built up, it seems likely that the lack of reflexivity and lack of attention to limitations of one’s own study, which are reported as outcomes of the content analysis of the articles in the ‘self-identified’ category of M&S’s sample, is a biased results of the content analysis’ lack of attention to the sections on methods and materials.

Secondly, there are problems regarding the subject matter categories of M&S’s content analysis (categories 1–5). The categories, ‘people concrete’, ‘people abstract’, ‘society’, ‘sex differences’ and ‘other statements’, chosen ‘with the intention to give a broad overview of the article content’ (Madison and Söderlund 2018: 1167) are generally very abstract. Compared to a full qualitative peer review for which I argued above, these categories are in their abstractness likely to dilute the content of the articles to such a degree that the basis for appropriately addressing the issue of scholarly quality is equally diluted.

Most importantly, category 4, ‘sex differences’, which should reflect a core content element of the articles, in so far as the articles of the sample are selected for their focus on gender and sex, does not seem to work well in the analysis. M&S legitimate their choice of ‘sex differences’ as subject matter category, arguing that they use ‘sex rather than “gender”’, ‘because virtually all these statements [the sex difference statements in the material, NL] are based on differences between males and females, rather than some index of social sex such as masculinity/femininity’ (Madison and Söderlund 2018: 1167). However, this argument demonstrates the authors’ lack of knowledge and understanding of the ways in which the key categories sex and gender are used in gender studies. The category ‘sex differences’ resonate with what in Ganetz’ terminology is termed ‘sex variable research’ (Ganetz 2005: 13), i.e. research where the categories women and men are used as variables, but where gender perspectives are not used as analytics to problematize the different ways in which gender is done, socially, culturally and in relation to biological bodies. Against this background, it is predictable that Figure 1 (Madison and Söderlund 2018: 1170) shows that the ‘self-identified’ article group scores particularly low regarding statements in this category (under 5% of the statements). It is more interesting to note that even the ‘neutral group’ does not seem to address this category much, even though it (also, for the reason indicated above, predictably) scores higher (between 5 and 10% of statements) than the articles in the ‘self-identified’ group.

However, that the ‘sex differences’ category shows these relatively low percentages (under 10% of the statements in all groups) is a problem for the validity of the analysis. Since the articles are chosen for their focus on sex and gender issues, it would be a reasonable expectation that the categories, included in the content analysis, somehow reflected this. However, the category ‘sex differences’ is evidently not doing the job well, and this means that the content analysis lacks a content category which can adequately capture potential differences or similarities regarding the core content of the articles.

Thirdly, it is to be noted that the construction of the quality indicator categories regarding ‘level of support’ (categories 6–10) consciously ‘ignored’ important clusters of statements, i.e. statements related to ‘scarcity of earlier studies’ and ‘reports of own results’ (Madison and Söderlund 2018: 1168). That these two kinds of statements were left out, when the categories for the content analysis were chosen, biases the analysis in disfavour of both gender studies and qualitative research. Gender studies was a relatively new research field, institutionalizing itself more broadly in the period under scrutiny in SGSL, 2000–2011. Therefore, it is to be expected that there, for a lot of the gender studies research reported on from this period, is a scarcity of earlier studies to refer to, and that a researcher sometimes may be the only one pursuing a certain set of questions and issues. These are given conditions for any new field of research and, certainly, a factor which needs to be taken seriously into account in a quality assessment. Added to this, it is to be noted that qualitative social science and humanities research, by contrast to quantitative social and natural science research, is not characterized by endeavours to obtain broad representativity, but is instead aimed at in-depth studies of specific local phenomena. Again, this implies that researchers, undertaking this kind of research, more often will tend towards being the only ones pursuing a certain set of questions and, therefore, in the previous research section of the article by nature of the research done, must refer to own results as a primary source of knowledge, and also be likely to mention scarcity of other studies in the field. Research, based, on anthropological fieldwork can serve to illustrate the point that this is a broad issue, relevant in quality assessments far beyond evaluations of the field of gender studies.

The lack of analysis of the methods and materials sections, the abstract subject matter categories, missing out on a category which can adequately address the core content of the articles, and the ‘level of support’ categories' way of leaving out statements related to ‘scarcity of earlier studies’ and ‘reports on own results’, are all factors which raise serious questions regarding the validity of M&S’s analysis. Against this background, it does not make sense to draw any other conclusions of the analysis than noticing that statistical differences emerge between the three article groups. But this indication of difference between different approaches to doing research on the issue of sex and gender is a rather predictable and unsurprising result, given the difference between sex variable research and gender studies research/research with gender perspective, which Ganetz (2005), based on a valid and reliable analytical framework, built up against the background of expert knowledge of the field, identified in her study of Swedish research council applications with gender markings.

## Conclusions

In the comment, I have discussed and identified a series of problems in the article of M&S—pertaining both to the overall conceptual framework, the general approach and the specific analysis. Firstly, I have demonstrated how the key concept of ‘gender perspective’ is used in a problematic and confusing way by M&S, which begs critical questions regarding their use of their alleged source of inspiration (Ganetz 2005). Secondly, I have indicated that the confusion occurring, when M&S borrow this key concept from Ganetz (2005), is not an isolated definitional problem, but related to a series of serious slippages and twists, which happen unacknowledged, when M&S draw on Ganetz’ conceptual framework and the implied methodology to divide their sample of articles into groups, relevant for the comparisons and quality assessment they want to make. Thirdly, with a starting point in the argument that the slippages occur due to the ways in which M&S try to transfer analytical tools from Ganetz’ qualitative study, based on a peer review methodology, which requires expert knowledge, to their own type of quantitative quality assessment, which they claim to be able to carry out without field specific expert knowledge, I have reflected on differences between qualitative and quantitative research—and more generally addressed difference between hermeneutic and explanatory knowledge production. I argued that for a quality assessment to be adequate, it would need to respect these differences, and develop its tools accordingly, which M&S’s analysis, as I have demonstrated it, fails to do. Fourthly, I have questioned the validity of the content analysis—the core of M&S’s study—against the background of categories used in the analysis as well as elements excluded from the analysis.

In conclusion, I shall return to the two main assumptions, on which M&S's article is based, (1) that Gender Studies can be defined as a bounded object to be quality assessed through a quantitative analysis, and (2) that a quality assessment of this academic field of research can be carried out without involving expertise from the field in question. My analysis of the problems, which I have identified in the article, suggests that, in different ways, they are related to the authors’ rejection of the need to reflect on and rethink these two basic assumptions—a rejection which the authors, as documented in the introductory section of my comment, firmly and explicitly stated already in their response to earlier criticism.

Nevertheless, I shall encourage the authors to rethink these assumptions. There are big problems regarding the validity of the present study—problems which, as my analysis has shown, stem both from the demonstrated lack of field-specific expertise involved in the study, and from the ways in which it tries to impose criteria from quantitative research and methods for measurement on a sample of articles which, it is to be assumed, is mixed in terms of representing both quantitative and qualitative research. In addition, if we cut away M&S’s (towards quantitative social and natural science biased) interpretations of what counts as scientific quality, and look at the analytic outcomes with the figures and statistics alone as our lens, then I can only conclude that the results are so predictable that the analysis in principle does not bring anything new. I do, as mentioned, not have access to the list of 36 articles, so I can only guess about the ways in which they are distributed with regard to representation of quantitative and qualitative studies. However, based on my knowledge of the field of gender studies and also on sex variable research (as well as on the list of research area of the first author of the journal articles (Table 1, Madison and Söderlund 2018: 1167), I find it reasonable to assume that the articles in the ‘self-identified’ and ‘inferred’ groups to a large extent are based on qualitative social science and humanities research, while, at least some of the articles, in the ‘neutral’ group represent sex variable research based on quantitative approaches. If this assumption on my part holds true, then it is a predictable conclusion that the latter group will come out as *different*—and if the quality indicators are *biased towards criteria for quality in quantitative research*, it is also rather predictable that *this bias will be reproduced in the outcomes,* so that the article group with most quantitative sex variable research will exhibit more ‘quality’ than the ones representing more qualitative research.

## Footnotes

1 All quotes from Ganetz (2005) are translated from Swedish into English by this author, NL.
